# Shifting sands in Saudi Arabian healthcare system towards value-based care: navigating the changing landscape

**DOI:** 10.3389/fpubh.2026.1765428

**Published:** 2026-03-18

**Authors:** Ahmed Al-Jedai, Hajer Almudaiheem, Wejdan Ibrahim Said Aburas, Eid Almutairi, Maysa Tariq Eshmawi, Khalid Al Sulaiman, Essam Tawfik, Mohammed Al-Luhidan, Mohammad Alowairdhi, Nancy Awad, Rita Ojeil, Mohammed Ahmed Alshennawi

**Affiliations:** 1 Colleges of Medicine and Pharmacy, Alfaisal University, Riyadh, Saudi Arabia; 2 Clinical Pharmacy, The Saudi Society of Clinical Pharmacy, Riyadh, Saudi Arabia; 3 Ministry of Health, Riyadh, Saudi Arabia; 4 King Faisal Specialist Hospital and Research Centre, Riyadh, Saudi Arabia; 5 College of Medicine, Imam Mohammed Ibn Saud Islamic University, Riyadh, Saudi Arabia; 6 King Abdullah Medical College Complex, Ministry of Health, Jeddah, Saudi Arabia; 7 King Abdulaziz Medical City, Riyadh, Saudi Arabia; 8 King Abdulaziz City for Science and Technology, Riyadh, Saudi Arabia; 9 Saudi Health Council, Riyadh, Saudi Arabia; 10 Saudi Food and Drug Authority, Riyadh, Saudi Arabia; 11 Pharmaceutical Sciences, Carexso, Dubai, United Arab Emirates; 12 Life Sciences Solution, PDC-CRO, Dubai, United Arab Emirates

**Keywords:** health technology assessments, Kingdom of Saudi Arabia, public-private partnership, Saudi Ministry of Health, Saudi Vision 2030, value-based healthcare

## Abstract

The KSA’s healthcare system is undergoing substantial transformation under Vision 2030, with a strategic shift toward value-based healthcare (VBHC). This perspective paper examines the national reform initiatives and implementation readiness using five interrelated domains: data interoperability and governance, health technology assessment (HTA) capacity and availability of local data, policy fragmentation across high-cost therapeutic areas, operational burden of managed entry and risk-sharing agreements, and equity and stakeholder trust. Evidence was synthesized from peer-reviewed literature, policy documents, and institutional sources from the Saudi Ministry of Health. The analysis highlights progress in digital health integration, formulary modernization, pharmacoeconomic evaluation, real-world data infrastructure, and expanding use of value-linked reimbursement mechanisms. However, persistent challenges include fragmented digital systems, limited technical capacity for advanced HTA, heterogeneous policy implementation in specialized therapeutic areas, administrative complexity associated with performance-based agreements, and ongoing equity and trust considerations. Emerging methodological advances, including cost-effectiveness thresholds, development of multi-criteria decision analysis frameworks, and establishment of a Saudi-specific EQ-5D-5L valuation, strengthen the foundation for VBHC implementation. In this paper, we have recommended an implementation-oriented assessment of system enablers and constraints and identifies priority areas to support scalable, evidence-based, and sustainable adoption of VBHC in the KSA.

## 1 Introduction

The healthcare system in the Kingdom of Saudi Arabia (KSA) is undergoing substantial reform under Vision 2030, with a strategic emphasis on improving the quality, efficiency, and sustainability of care delivery. Healthcare is recognized as a fundamental right under the Basic Law of Governance, and the Ministry of Health (MoH) remains the primary steward of a predominantly publicly financed system providing universal access to healthcare services (1–4). Within this reform agenda, the Health Sector Transformation Program has articulated a shift toward patient-centered, outcome-oriented care models supported by digital transformation and evidence-based decision-making (5–8).

Over the past decade, several reviews have documented the broad impact of Vision 2030 on the KSA’s healthcare system, highlighting progress in governance, service delivery models, and system strengthening (9–16). While these studies provide valuable descriptive insights, they largely emphasize policy intent and strategic alignment, with limited critical assessment of implementation feasibility, methodological rigor, or system readiness for value-based healthcare (VBHC) adoption. In particular, the operational role of health technology assessment (HTA), real-world evidence (RWE), and value-linked reimbursement mechanisms have not been examined in sufficient detail, particularly in relation to high-cost therapeutic areas such as cancer, rare diseases, etc. that pose persistent challenges due to clinical heterogeneity, limited local evidence at the time of market entry, and substantial budget impact.

The conceptual foundation of VBHC remains contested in academic literature. Porter originally defined value as health outcomes achieved per dollar spent, emphasizing condition-specific care cycles, provider competition, and bundled reimbursement mechanisms (17). In contrast, population-health scholars argue that value must incorporate broader system-level considerations, including equity, prevention, and social determinants of health, rather than solely episode-level cost-outcome optimization (18).

Saudi Arabia’s reform trajectory reflects elements of both interpretations. While procurement modernization and managed-entry agreements (MEAs) align with Porter’s micro-level value optimization framework, Vision 2030’s emphasis on prevention, digital health, and universal access, signals a broader population-health orientation. The coexistence of these paradigms introduces important questions regarding how “value” is defined, measured, and operationalized within the Saudi context.

VBHC reframes healthcare performance around optimizing patient-relevant outcomes relative to costs, rather than the volume of services delivered (19). Most VBHC frameworks have been developed in decentralized or pluralistic payer environments, supported by mature data infrastructures and long-established HTA institutions. The adoption of VBHC model has gained increasing prominence both nationally and globally in response to rapidly rising healthcare costs and growing budgetary constraints (20, 21). Evidence from centrally financed healthcare systems, including those in the Middle East, remain comparatively limited and fragmented.

In the KSA, healthcare expenditure continues to rise, driven in part by demographic changes, increasing prevalence of chronic diseases, and growing adoption of high-cost pharmaceuticals and advanced medical technologies. Healthcare cost is expected to reach US$135.5 billion by 2027, compared with US$74.7 billion in 2017 (22), while per capita healthcare expenditure increased from US$1,198 in 2015 to US$1,442 in 2021 (23). Pharmaceuticals and medical technologies account for approximately 20% of total healthcare expenditure, underscoring the need for structured value-based prioritization and sustainable reimbursement mechanisms (24).

In light of these challenges, the MoH has introduced several initiatives aligned with VBHC principles, including modernization of formulary decision-making processes, expanded use of pharmacoeconomic evaluations, development of disease registries, and increasing adoption of MEAs and risk-sharing agreements (RSAs). These initiatives aim to balance patient access to innovative therapies with financial sustainability, particularly in high-cost therapeutic areas (25, 26). However, the extent to which such tools can be scaled and institutionalized within a centralized public payer system remains uncertain (27). Accordingly, we conducted a comprehensive examination of VBHC implementation in the KSA. Our analysis concentrates on five interrelated domains central to operationalizing VBHC in a centrally financed system: (1) data interoperability and governance, (2) HTA capacity and availability of local data, (3) policy fragmentation across high-cost therapeutic areas, (4) operational burden of value-based and risk-sharing agreements, and (5) equity considerations and stakeholder trust. By examining these domains, the review seeks to contribute a critical and implementation-oriented perspective to the evolving discourse on VBHC in Saudi Arabia.

This paper advances the literature by moving beyond descriptive reform summaries to provide an analytical assessment of Saudi Arabia’s transition toward VBHC. Specifically, it examines the institutional instruments deployed to operationalize value, evaluates early implementation signals and governance challenges, and situates the Saudi experience within broader theoretical debates on value definition, outcome measurement, and fiscal sustainability.

## 2 Methods

We adopted a narrative review approach, with structured evidence mapping. The objective was not to perform an exhaustive systematic synthesis but to provide an interpretive and policy-oriented analysis of key domains relevant to the implementation of VBHC in Saudi Arabia.

To enhance methodological transparency, a structured but non-systematic literature search was undertaken to identify relevant peer-reviewed and institutional sources informing the five analytical domains examined in this review.

The primary database consulted was PubMed. Searches were conducted using combinations of keywords and boolean operators, including: ((“value-based healthcare” OR “value-based care”) AND “Saudi Arabia”) OR ((“health technology assessment” OR HTA) AND “Saudi Arabia”) OR ((“managed entry agreements” OR MEA OR “risk-sharing agreements”) AND “Saudi Arabia”) OR ((“real-world evidence” OR RWE OR “electronic health records”) AND “Saudi Arabia”). Searches were limited to English-language publications between 2010 and 2025 to align with the timeline of major health system reforms under Vision 2030. Evidence sources included national policy documents, peer-reviewed literature, and institutional materials, including gray literature from World Health Organization (WHO) and the Organization for Economic Co-operation and Development (OECD), and national authorities.

Inclusion criteria comprised peer-reviewed publications and institutional documents that addressed VBHC implementation, HTA, reimbursement mechanisms, RWE infrastructure, or health system reform in Saudi Arabia or comparable centralized healthcare systems. Titles and abstracts were screened for relevance to system-level implementation of VBHC, HTA capacity, reimbursement mechanisms, real-world data (RWD) infrastructure, and policy governance in Saudi Arabia or comparable centralized healthcare systems. Studies were excluded if they were purely opinion-based without policy or methodological relevance, duplicated previously indexed material, lacked sufficient methodological transparency, or were not directly related to system-level implementation considerations.

Gray and institutional documents were also screened for reliability by restricting inclusion to materials published by recognized governmental bodies, regulatory authorities, international organizations (e.g., WHO, OECD), or peer-reviewed professional organizations. Where possible, information from gray sources was cross-checked against multiple public sources or peer-reviewed literature to ensure consistency and credibility.

Full texts of potentially relevant articles were reviewed to assess conceptual and policy relevance. Given the narrative nature of the review, formal risk-of-bias assessment was not performed. Instead, priority was given to peer-reviewed empirical studies, systematic reviews, and official policy documents with clearly articulated methodological foundations. In total, 756 records were screened, of which 93 sources were deemed directly relevant and included in the final synthesis. These comprised 75 full-text peer-reviewed journal articles and 18 institutional or website-based sources, including national policy documents, governmental publications, and materials from recognized international organizations. Evidence was synthesized using a thematic analytical approach. The five analytical domains (data interoperability and governance; HTA capacity and availability of local data; policy fragmentation across high-cost therapeutic areas; operational burden of value-based agreements; and equity and stakeholder trust) were predefined based on established VBHC frameworks and contextual policy priorities in Saudi Arabia. Identified evidence was mapped to these domains to enable structured comparison and interpretation. Where multiple types of sources addressed similar issues, interpretations were informed primarily by peer-reviewed empirical research, while institutional documents were used to contextualize national strategies and implementation mechanisms.

Given the inclusion of both peer-reviewed literature and institutional materials, distinctions were made between empirical evidence, policy documentation, and expert commentary. Conclusions regarding system readiness and implementation constraints were derived primarily from peer-reviewed studies and methodologically robust reports. Institutional documents were used to describe formal policy positions and reform trajectories but were not treated as independent empirical validation. As a narrative review, this study does not claim exhaustive coverage of all relevant literature but aims to provide a structured, policy-oriented synthesis to inform scholarly and institutional discussion.

## 3 Implementation of VBHC in KSA: enablers and challenges

### 3.1 Data interoperability and governance

Despite considerable advancement in healthcare reform, persistent system-level constraints continue to influence the pace and effectiveness of VBHC implementation in the KSA. One of the most prominent challenges relates to the fragmentation of digital health infrastructure and the lack of unified electronic health records (EHRs). Fragmented EHR systems across public and semi-autonomous institutions present challenges in managing patient information efficiently, limiting longitudinal patient tracking and complicating outcomes measurement across the healthcare continuum (28, 29). This fragmentation directly affects the generation and use of RWD essential for VBHC.

RWD and RWE are core components of VBHC, particularly in therapeutic areas where randomized controlled trial data are scarce (30, 31). Building a resilient RWD/RWE framework requires interoperable digital health systems, strong governance structures, and coordinated collaboration among stakeholders. In this context, disease registries and digital health platforms have expanded significantly enabling improved data capture for regulatory compliance, pharmacovigilance activities, and value-based pricing models. Scaling disease registries, especially for rare conditions, and leveraging the digital advancements already present in the KSA is critical to producing actionable and context-specific evidence (30).

The establishment of the Saudi Cancer Registry in 1992 marked a pioneering step in disease registry development with its statistical data guiding cancer care and prevention strategies over the past three decades (32), followed by continued efforts by the Saudi Health Council (SHC) and the MoH toward broadening and updating the scope and applicability of the existing disease registries across disease areas such as sickle cell anemia, multiple sclerosis, bleeding disorders, respiratory, and other rare and genetic diseases (33–35).

However, a recent study has highlighted challenges in data accuracy, completeness, and reliability, including difficulties in gathering comprehensive and accurate data due to fragmented sources and inconsistent methods. Governance challenges such as privacy concerns, data sharing policies, and regulatory frameworks, further complicate the effective use of RWD for VBHC decision-making (36).

### 3.2 Health technology assessment capacity and availability of local data

HTA has increasingly been integrated into value-based decision-making processes in the KSA (37). The MoH has established platforms to support evidence-based formulary decision-making, pharmacoeconomic evaluation, and RWE generation. These efforts aim to strengthen value optimization within the healthcare system by aligning clinical benefit with economic sustainability.

A central component of this approach is the enhanced formulary inclusion process overseen by the Pharmacy and Therapeutics (P&T) committee, operating under the umbrella of MoH. The current process involves the assessment of five criteria: comprehensive drug information (dose, indication, and route of administration), safety evidence, published clinical efficacy data, pharmacoeconomic analyses, and local budget impact analysis (BIA) (38, 39).

Pharmacoeconomics plays a crucial role in these decisions; however, an effective application requires sufficient expertise among healthcare professionals and hospital administrators. To this end, there is a recognized need to increase education and recruitment of pharmacoeconomic experts to optimize the management of limited healthcare resources (38).

Economic evaluation has become an essential tool for the assessment, validation, and comparison of outcome benefits and costs among different products or interventions, enabling value-based decision making. These analyses can inform reimbursement frameworks, price negotiations, and the development of clinical practice guidelines and policies (40, 41). To date, numerous economic evaluations have been successfully conducted across various therapeutic areas, adhering to international guidelines and best practices (42). These evaluations typically involve collaboration among P&T committee members, clinical experts, and third-party vendors, including pharmaco-economists, subject matter experts, and model developers, and have contributed to payer and policy decisions with substantial potential for cost optimization and healthcare resource efficiency (43).

In the KSA, there has been a notable shift in the commonly used economic evaluation methodology. While BIA has traditionally been used more (44, 45), there has been a gradual shift toward more value-based evaluations, particularly cost-effectiveness analysis (CEA) (46).

Despite this progress, the robustness of HTA outputs remains constrained by the limited availability of high-quality local epidemiological, cost, and utility data. Consequently, many economic models rely on external inputs or assumptions, underscoring the need for stronger collaboration between health economic researchers, policymakers, and healthcare institutions to generate locally relevant evidence (43).

### 3.3 Policy fragmentation across high-cost therapeutic areas

Policy fragmentation remains a key challenge in the implementation of VBHC across high-cost therapeutic areas in the KSA. Demographic changes, including population growth, improved life expectancy, high birth rate and rapidly aging population, have intensified demand for healthcare services. The life expectancy increased to ~75 years in 2020 and is expected to rise even further to ~80 years by 2050, while the population aged >60 years is expected to reach 9.5% by 2035 and 18.5% by 2050 (47). According to the United Nations, the total population is projected to grow from over 34 million to 54.7 million by 2050 (4, 48), placing additional strain on healthcare infrastructure and financing.

High-cost therapies, particularly in complex disease areas, are managed through a combination of national programs and decentralized decision-making pathways. While some disease areas benefit from structured policies and centralized oversight, others experience variability in evidence requirements, access criteria, and reimbursement mechanisms. This heterogeneity limits the consistent application of value-based principles and complicates long-term planning and prioritization (25, 26).

Financial strain on the national healthcare budget further exacerbates these challenges, driven by rising costs of medical supplies, advanced medical technologies, and workforce expansion (49). Structural and policy-level fragmentation across institutions may further contribute to uneven access and inefficiencies, underscoring the need for improved alignment and harmonization across high-cost therapeutic areas (43).

These observed disparities raise fundamental normative questions regarding how ‘value’ is operationalized within the Saudi health system. While VBHC frameworks often prioritize efficiency metrics such as cost per QALY gained (50), distributive justice scholarship emphasizes that healthcare allocation must also reflect principles of fairness, equity, and priority to disadvantaged populations. In contexts characterized by geographic variation and differential access between citizens and expatriates, reliance solely on cost-effectiveness metrics may risk reinforcing existing inequities unless accompanied by explicit equity safeguards (51, 52).

### 3.4 Operational burden of value-based and risk-sharing agreements

MEAs and RSAs have been increasingly adopted by the MoH as mechanisms to support access to innovative therapies while managing financial and clinical uncertainty. MEAs serves as reimbursement tools that distribute financial risk between manufacturers and payers, addressing uncertainties related to the real-world clinical effectiveness, potential benefits, and cost-effectiveness by following a “value for money” approach (53). MEAs are categorized into financial-based agreements (FBA) and value-based agreements (VBA), with hybrid models combining both approaches (54).

MEAs introduce financial risk-sharing mechanisms that complement HTA by managing uncertainty in real-world performance; however, their administrative complexity may impose governance strain, particularly in systems where registry integration and outcome tracking remain under development, The simultaneous expansion of VBAs, CET adoption, and registry digitization raises coordination challenges across procurement, clinical, and analytical units. Without clear delineation of institutional responsibilities, and data governance protocols, reform layering may generate transaction costs that offset intended efficiency gains (19).

In the KSA, while FBAs remains more prevalent due to relative simplicity offering immediate gains to stakeholders (54), VBAs tie costs to clinical value, introducing complexities in long-term monitoring and data collection. The MoH has implemented 5 VBAs with medical product manufacturers (26). These agreements often incorporate complementary services such as additional devices and goods, disease awareness initiatives, educational programs, and comprehensive patient support services to enhance therapeutic value (26, 55).

However, VBAs introduce significant operational complexity. Defining measurable outcomes, ensuring data availability, monitoring performance over time, and managing contractual obligations impose administrative burdens on payers, providers, and manufacturers. Although structured implementation frameworks have been established—comprising internal assessment, early dialog, formal negotiations, and contract implementation—stakeholders continue to face challenges related to data generation and administrative capacity (54, 55). These operational considerations affect the scalability and sustainability of performance-based agreements within a centralized public payer system. Table 1 presents example case studies highlighting the successful implementation of VBA in the healthcare system of the KSA (56–60).

**Table 1:** Case studies illustrating the advantage of adopting budget impact analysis (BIA) and cost-effectiveness analysis (CEA), and successful implementation of value-based agreements.

Study focus/condition	Description	Outcome/implications	References
Budget impact analysis			
Emicizumab for hemophilia A (HA)	Evaluation in the KSA for HA patients indicated a significant 21.5% cost reduction over 5 years when transitioning from the standard of care. Costs included drug, administration, travel, surgeries, and hospitalization	Significant reduction in overall treatment costs for HA patients with inhibitors	(88)
Biologics for moderate-to-severe psoriasis	Introduction of biologics (ixekizumab, guselkumab, risankizumab, and/or secukinumab) led to a minimal 0.4–0.8% increase in the MoH budget over 5 years. This resulted in a Value-Based Agreement (VBA) for free initial treatment and outcomes-based reimbursement	Minimal budget impact; initiated a VBA to reduce costs and improve patient access	(89)
PCSK9 inhibitors for familial hypercholesterolemia	Implementing PCSK9 inhibitors led to a 6.1% annual increase in budget (SAR 91.16 million) but decreased cardiovascular events offset costs slightly. This led the MoH to restrict PCSK9 inhibitors use to high-risk patients and initiate a VBA for better access	Formulary inclusion with restricted use; VBA initiated to offset increased budget costs	(90)
Biologics for Crohn’s disease and ulcerative colitis	Adding biologics like vedolizumab led to only marginal cost increases	Vedolizumab added to formulary with minimal cost impact	(91)
Cost-effectiveness analysis			
Multiple sclerosis (MS) treatment options	Cost-effectiveness analysis for MS treatments (fingolimod, teriflunomide, etc.) identified Rebif as optimal at a willingness-to-pay (WTP) of $100,000, but none were cost-effective under this amount. Avonex had the lowest ICER of $337,282 per QALY	Findings highlight the need for higher WTP thresholds to achieve cost-effectiveness in disease-modifying drugs (DMDs) for MS	(92)
Value-based agreements			
Multiple sclerosis	MoH implemented a VBA with free treatment initiation and MRI-based outcome assessments. This reduced progression, relapses, and expedited access to optimal treatments	VBA enabled risk-sharing and better alignment of cost–benefit	(56, 57)
Spinal muscular atrophy (SMA)	Due to limited treatment and cost concerns, MoH established a VBA with cost-sharing, free medication for a percentage, and a reimbursement model based on patient outcomes. Additionally, gene therapy costs are spread over 5 years with reimbursement linked to outcomes	VBA promoted access and supported evidence-based use of gene therapy for SMA	(58, 93)
Psoriasis treatment (Risankizumab, Ixekizumab)	High cost and limited real-world evidence prompted VBAs, improving access and contributing to real-world evidence generation	VBA alleviated financial burden while addressing evidence gaps	(59, 93)
Hypercholesterolemia (Evolocumab)	For cost and effectiveness uncertainties, MoH implemented a VBA for evolocumab, aimed at addressing challenges with LDL reduction in high-risk patients	VBA supported cost management while offering access to promising treatments for high-risk patients	(60)

This table is compiled by the authors based on published literature and publicly available reports.

Taken collectively, the case studies presented in Table 1 illustrate a consistent policy logic underpinning Saudi Arabia’s VBHC implementation; economic evaluation is not deployed in isolation but embedded within adaptive reimbursement strategies. Budget impact analyses are frequently used as an initial fiscal screening tool, particularly in high-prevalence or high-cost conditions, while cost-effectiveness evaluations inform longer-term value assessment. Where uncertainty persists, managed entry agreements serve as a traditional mechanism to record innovation access with fiscal prudence.

This layered deployment of economic tools suggests a pragmatic sequencing strategy rather than rigid adherence to a single methodological framework. The Saudi approach appears to combine short-term budget containment with conditional access arrangements, thereby managing both fiscal risk and evidentiary uncertainty.

### 3.5 Equity considerations and stakeholder trust

Equity considerations remain central to the successful implementation of VBHC in the KSA. Despite universal healthcare coverage, inequities persist in healthcare access and resource allocation, particularly between urban and rural regions and between services available to citizens versus expatriate populations. Workforce shortages and uneven distribution of healthcare facilities exacerbate these disparities, contributing to longer waiting times, increased patient loads, and potential compromises in care quality and patient safety (61).

The growing burden of chronic diseases such as diabetes, hypertension, and cardiovascular conditions further increases demand for long-term healthcare resources and coordinated care delivery (62). Decision-support tools like multi-criteria decision analysis (MCDA) help promote fairness and transparency in health policy evaluation (63). However, meaningful patient involvement in HTA, reimbursement decisions, and policy development remains limited.

KSA has instituted several initiatives aimed at improving equity, access, and patient-centered care that are relevant to these concerns. Under Vision 2030 and the Health Sector Transformation Program, reforms emphasize expanding access through increased primary care services, preventive health, and integrated care models designed to reduce regional disparities in service availability and outcomes. These reforms also include investments in telemedicine and mobile health services to reach underserved and remote populations, and the development of health clusters intended to decentralize care and improve service coordination across regions (64, 65).

To address chronic disease management and proactive patient engagement, digital health innovations—such as the establishment of a national chronic disease command center for diabetes—have been introduced to support real-time monitoring and early intervention, reflecting a commitment to preventive care and systematic management of high-burden conditions (66).

Strengthening structured ethical review processes and incorporating patient advocacy perspectives into decision-making frameworks could enhance stakeholder trust and improve the legitimacy of value-based healthcare policies (67). Addressing equity and trust alongside technical and operational considerations is essential for ensuring that VBHC reforms are both sustainable and socially acceptable within the Saudi healthcare system.

## 4 Future agenda for the advancement of the VBHC model

**Figure 1:**
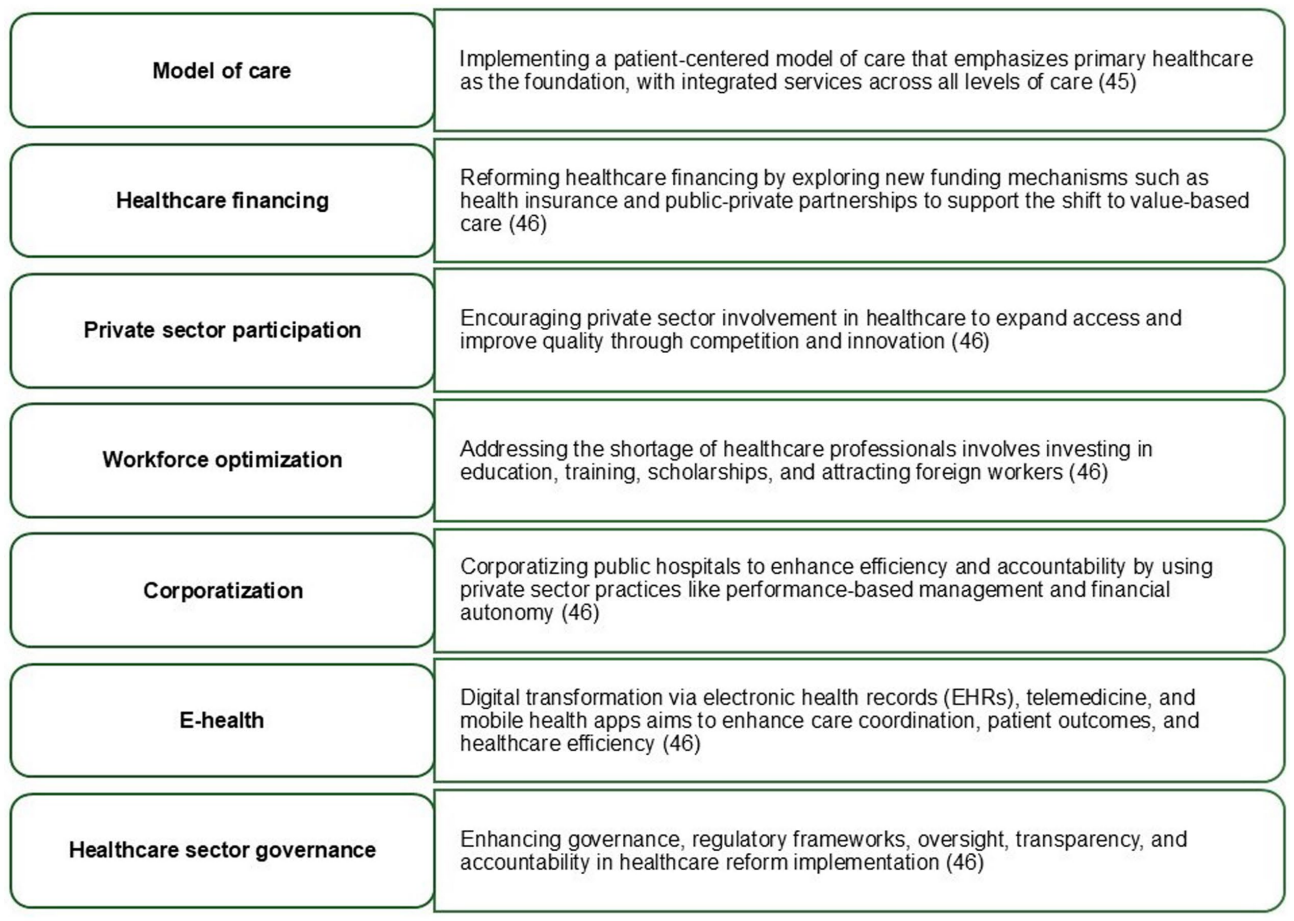
Seven key focus areas for a strategic transformation toward a value-based healthcare system in the KSA. This figure is author-constructed based on synthesis of publicly available national strategy documents and policy frameworks. Sullivan et al. (45) and Weinstein and Stason (46) indicate the primary source documents that informed the identification of the strategic focus areas.

Advancing the VBHC model in the KSA will require a coordinated strategic agenda that strengthens evidence generation, incentivizes quality outcomes, and modernizes system-wide governance. This approach has been gaining renewed impetus with innovative research, digital transformation of the healthcare system and increasing monetary constraints. The MoH is strategically addressing this transformation by determining seven key focus areas as mentioned in Figure 1 (45, 46).

Collectively, these reforms illustrate a multi-layered transformation toward VBHC; however, their systemic interaction warrants careful evaluation. Registry expansion, economic evaluation, MEAs, CET development, and digital infrastructure are mutually reinforcing but interdependent mechanisms. The sustainability of Saudi Arabia’s VBHC transition will therefore depend less on the breadth of reforms and more on their coordinated maturation, analytical coherence, and institutional capacity alignment.

### 4.1 Scaling integrated and digital models of care for rare diseases

The Saudi MoH has shown a strong commitment to improving healthcare quality by establishing centers of Excellence (CoE) across multiple therapeutic areas including rare diseases. These centers act as hubs of specialized expertise, providing comprehensive and cohesive care across the patient pathway, from diagnosis through long-term management. Following evidence-based guidelines, CoEs aim to standardize care delivery, optimize clinical outcomes, and promote innovation by consolidating expertise, infrastructure, and multidisciplinary collaboration at the national level (68).

Building on this foundation, the MoH is leveraging digital technologies to establish disease-specific digital care pathways tailored to rare diseases (69). These pathways provide standardized framework for diagnosis, treatment, and management supporting timely access to appropriate care. Integrated digital tools such as clinical decision support algorithms, remote monitoring technologies, and patient portals facilitate enhanced communication and collaboration between patients and healthcare providers. Collectively, these capabilities support improved disease understanding, treatment optimization, and patient engagement (70).

Digital infrastructure functions as a foundational enabler of VBHC; yet digital expansion alone does not guarantee outcome measurability or payment reform coherence. The sequencing of digital health adoption relative to payment reform is critical; premature implementation of outcome-contingent reimbursement without interoperable data systems risks contractual ambiguity and provide resistance. Thus, digital maturity must be evaluated not only in terms of technological deployment but also institutional integration.

To further enable system-wide scaling and data-driven decision-making the MoH has planned initiatives for launching a data registry for priority rare diseases diagnosed within the public health system. These registries will integrate remote patient management through electronic patient-reported outcomes (e-PROs), teleconsultation, and connected monitoring devices. In parallel, selected rare disease centers will be designated to standardize and streamline the patient’s journey from diagnosis to treatment. The planned integration of artificial intelligence is expected to support early diagnosis, improved linkage to treatment, and care optimization, thereby further advancing the quality and accessibility of care for patients with rare diseases (37).

While expansion of disease registries strengthens the evidentiary foundation for VBHC, their utility is contingent upon data completeness, interoperability, and methodological standardization. The increasing reliance on economic evaluation and outcome-based contracting presupposes robust epidemiological and cost data; yet persistent fragmentation and data quality limitations introduce epistemic risk into cost-effectiveness modeling. In this context, registry expansion and HTA institutionalization must be sequenced deliberately, ensuring that evidentiary infrastructure matures in parallel with reimbursement sophistication. Otherwise, VBHC mechanisms risk operating on analytically fragile foundations.

### 4.2 Establishing cost-effectiveness threshold, multi-criteria decision analysis, and EQ-5D-5L valuation

The continued development of HTA is anticipated to strengthen the methodological foundation of VBHC in the KSA. Although HTA is still in its early stages of institutionalization, national roadmaps and capacity-building activities have rapidly progressed, reflecting increasing alignment with international best practices (71, 72). Pharmacoeconomic analyses, including CEA or cost-utility analysis (CUA) represent core components of HTA and are central to transparent and evidence-based resource allocation.

The growing integration of CEA into formulary decision-making represents a methodological advancement; however, the robustness of these models depends critically on local epidemiological parameters, utility weights, and cost inputs. Where such data are incomplete or extrapolated from international sources, uncertainty may accumulate within decision models. This raises important questions regarding threshold calibration, opportunity cost estimation, and the ethical defensibility of resource allocation under evidentiary constraint. Strengthening local data generation must therefore be understood not as a parallel reform, but as a prerequisite for credible VBHC institutionalization.

The formal adoption of national cost-effectiveness threshold (CET) represents a critical next step for standardizing decision-making. Proposed central estimates ranging from SAR 50,000 to SAR 75,000 per quality-adjusted life-year (QALY) gained, would provide clearer benchmarks for reimbursement and investment decisions, supporting consistency, predictability, and in value-based prioritization (72). The operational relevance of CET implementation is further strengthened by the availability of locally derived health-state utility values, following the establishment of a nationally representative EQ-5D-5L value set using time trade-off and discrete choice experiment methods (73). The availability of Saudi-specific preference weights enhances the methodological validity of cost-utility analyses and reduces reliance on external value sets that may not reflect local population preferences. In parallel, the development of the MCDA framework aligns with broader policy objectives to incorporate clinical, economic, social, and equity considerations into priority setting under a VBHC model. Early implementation experience highlights the importance of stakeholder awareness, methodological standardization, and capacity-building to ensure consistent application. Additionally, establishment of national disease registries and therapy-specific databases for oncology, neurology, and autoimmune diseases will ensure robust evidence requirements for MCDA analysis (73).

Standardization of health-related quality-of-life measurements is also advancing in KSA. In 2021, the MoH, in collaboration with the EuroQoL group, initiated the development of EQ-5D-5L valuation instrument using time trade-off and discrete choice experiment methods to establish population-based preference weights for the Saudi population (74). The resulting national value set reduces reliance on foreign preference weights that may not adequately reflect local societal values and strengthens the methodological validity of utility estimates applied in economic models. The valuation exercise demonstrated a predicted utility score of 0.683 for the worst health state, providing an empirical basis for economic modeling in the Saudi context (75).

As adoption expands across healthcare providers, researchers, and policymakers, the national EQ-5D-5L value set will improve data harmonization, enhance the credibility of economic evaluations, and strengthen HTA capacity under the Vision 2030 health transformation agenda. Together, the establishment of CET benchmarks, maturation of MCDA frameworks, and national valuation of health utilities will support more transparent, consistent, and value-oriented decision-making across the Saudi health system.

## 5 Discussion

Saudi Arabia’s healthcare transformation under Vision 2030 has enabled considerable strides toward operationalizing VBHC through reforms in governance, procurement, digital infrastructure, and reimbursement frameworks. A range of strategic measures has reshaped regulatory processes, procurement models, and access pathways, supporting greater transparency, cost efficiency, and improved access to essential therapies. Public sector procurement is standardized through the National Unified Procurement Company’s (NUPCO) centralized tendering mechanism, enabling unified sourcing of pharmaceuticals and medical technologies, while private sector acquisition pathways remain more flexible, contingent on product authorization by the Saudi Food and Drug Authority (SFDA) (25). These procurement reforms, alongside broader investments in digital health integration and centralized purchasing modernization, reflect continued momentum under the Vision 2030 health reform agenda (76, 77).

At the policy level, the implementation of modernized formulary processes, pharmacoeconomic evaluations, and adoption of MEAs and RSAs, reflects increasing alignment with global best practices in value-oriented care (78, 79). However, despite clear prioritization in selected clinical domains, other areas—particularly those requiring specialized expertise or serving smaller patient populations—continue to experience fragmented policy development and variable implementation (80).

Vision 2030 further emphasizes prevention-oriented reform strategy supported by digital health innovation and strengthened emergency preparedness capabilities. National public health interventions targeting lifestyle and behavioral changes as well as school and community programs, aim to reduce the long-term burden of non-communicable diseases (81, 82). Additionally, the adoption of artificial intelligence driven diagnostic tools, EHRs, and telemedicine platforms has contributed to operational efficiency and expanded access to specialized services (30, 83). Strategic collaboration with the WHO has reinforced health emergency preparedness and health system resilience (84).

Operational examples illustrate how these systems reforms translate into measurable service improvements. The establishment of a specialty pharmacy within a state-supported tertiary hospital demonstrated improved medication availability, individualized counseling, more efficient dispensing workflows resulting in higher patient satisfaction, a 52% reduction in pharmacy waiting times, and improved adherence to specialty drugs (85). Such localized service innovations highlight the potential for operational efficiency gains and patient-centered improvements consistent with VBHC principles.

Structural fragmentation remains across specialized therapeutic areas, evolving technical capacity for advanced HTA methodologies, and limitations in data quality, privacy, and governance constrain the scalability of RWE applications (86). Cultural resistance to new healthcare models and practices, regional instability, and geopolitical tensions may divert institutional attention and resources (16, 39, 87).

VBHC implementation in the KSA is occurring within a complex political economy landscape characterized by centralized fiscal authority, public-sector dominance, and ongoing privatization reforms. The shift toward outcome-based reimbursement may alter financial flows across hospitals, pharmaceutical suppliers, and regional health clusters, potentially generating institutional resistance.

Additionally, as procurement transitions toward more performance-contingent contract, risk-distribution between government and manufacturers becomes a strategic negotiation arena rather than a purely technical assessment. Ensuring transparency in CET determination, MEA negotiation, and registry governance will be essential to maintaining stakeholder trust.

Long-term fiscal sustainability also depends on whether cost containment achieved through VBAs offsets the structural drivers of expenditure growth, including demographic, aging, epidemiological transition, and high-cost gene and precision therapies. Without parallel investments in prevention and primary care integration, VBHC risks becoming a sophisticated procurement mechanism rather than a fully integrated system reform.

Overall, the Kingdom’s trajectory reflects a health system transitioning from volume-driven care toward outcome- and value-oriented care. Strong policy commitment, continued digital investment, strengthened HTA capacity, and coordinated governance will be essential to institutionalize VBHC, reduce fragmentation, and achieve long-term improvements in equity, efficiency, and population health outcomes at scale.

## 6 Conclusion

KSA’s transition toward VBHC reflects a broader shift in how healthcare priorities are defined, justified, and governed within a rapidly transforming system. Progress to date demonstrates increasing institutional readiness to move beyond volume-driven decision-making. However, translating reform into routine practice will require consistent execution across organizations, clearer accountability structures, and sustained organizational learning. Ensuring that value principles are embedded into everyday operational decisions rather than remaining confined to pilot initiatives will be essential for long-term impact. Sustained institutional alignment, transparent governance, and implementation discipline, are critical to translating strategic reform into measurable improvements in population health outcomes and system sustainability.
